# Breast cancer screening and early diagnosis in Chinese women

**DOI:** 10.20892/j.issn.2095-3941.2021.0676

**Published:** 2022-04-05

**Authors:** Rui Ding, Yi Xiao, Miao Mo, Ying Zheng, Yi-Zhou Jiang, Zhi-Ming Shao

**Affiliations:** 1 Key Laboratory of Breast Cancer in Shanghai, Department of Breast Surgery, Fudan University Shanghai Cancer Center, Shanghai 200032, China; 2 Department of Oncology, Shanghai Medical College, Fudan University, Shanghai 200032, China; 3 Department of Cancer Prevention, Fudan University Shanghai Cancer Center, Department of Oncology, Shanghai Medical College, Fudan University, Shanghai 200032, China; 4 Precision Cancer Medicine Center, Fudan University Shanghai Cancer Center, Shanghai 200032, China

**Keywords:** Breast cancer, screening, Chinese, imaging screening, genetic test

## Abstract

Breast cancer is the most common malignant tumor in Chinese women, and its incidence is increasing. Regular screening is an effective method for early tumor detection and improving patient prognosis. In this review, we analyze the epidemiological changes and risk factors associated with breast cancer in China and describe the establishment of a screening strategy suitable for Chinese women. Chinese patients with breast cancer tend to be younger than Western patients and to have denser breasts. Therefore, the age of initial screening in Chinese women should be earlier, and the importance of screening with a combination of ultrasound and mammography is stressed. Moreover, Chinese patients with breast cancers have several ancestry-specific genetic features, and aiding in the determination of genetic screening strategies for identifying high-risk populations. On the basis of current studies, we summarize the development of risk-stratified breast cancer screening guidelines for Chinese women and describe the significant improvement in the prognosis of patients with breast cancer in China.

## Introduction

Breast cancer is the most common malignant tumor worldwide^1^. In China, the incidence of female breast cancer continues to increase, thus endangering women’s health^2^. Breast cancer screening is an important method for secondary prevention. It facilitates early detection, diagnosis, and treatment of breast cancer, and effectively improves patient survival and prognosis^3,4^. Western countries began to standardize the breast cancer screening process earlier than China and have already established many screening guidelines for breast cancer^5–13^. However, the screening rate in women in China is much lower than that in Western countries, and a national screening program in China is difficult to establish^14^. Additionally, breast cancer shows clear differences according to ancestry: Chinese women have unique breast cancer risk factors, breast structure features, and characteristics of breast cancer onset^15,16^. Therefore, standard evidence-based screening guidelines suitable for Chinese women are needed to guide and promote breast cancer screening in China.

In this review, we systematically discuss the epidemiological and population characteristics of breast cancer in China. The risk factors and genetic features of Chinese patients are summarized. Furthermore, we elucidate the establishment of screening guidelines suitable for Chinese women.

## Increasing incidence of breast cancer in China

### Breast cancer incidence and mortality in China

Breast cancer incidence and mortality have increased in recent years. According to the Global Cancer Observatory (GLOBOCAN) 2020 estimates of cancer incidence and mortality, female breast cancer has become the most commonly diagnosed cancer and the fifth leading cause of death among all cancers, with an estimated 2.3 million new cases and 685,000 deaths per year^1^. Among women, breast cancer accounts for 1 in 4 cancer cases and causes 1 in 6 cancer-related deaths. Globally, 1 in 18 women develops breast cancer in her lifetime^17^. Breast cancer also substantially influences Chinese society. GLOBOCAN 2020 included morbidity and mortality information for breast cancer from 23 Chinese cancer registries. In China, breast cancer is the cancer type with the highest incidence in women, with 416,371 new cases and 117,174 deaths in 2020, accounting for 18% of new cases and 17% of deaths worldwide, respectively^18^.

**Figure 1:**
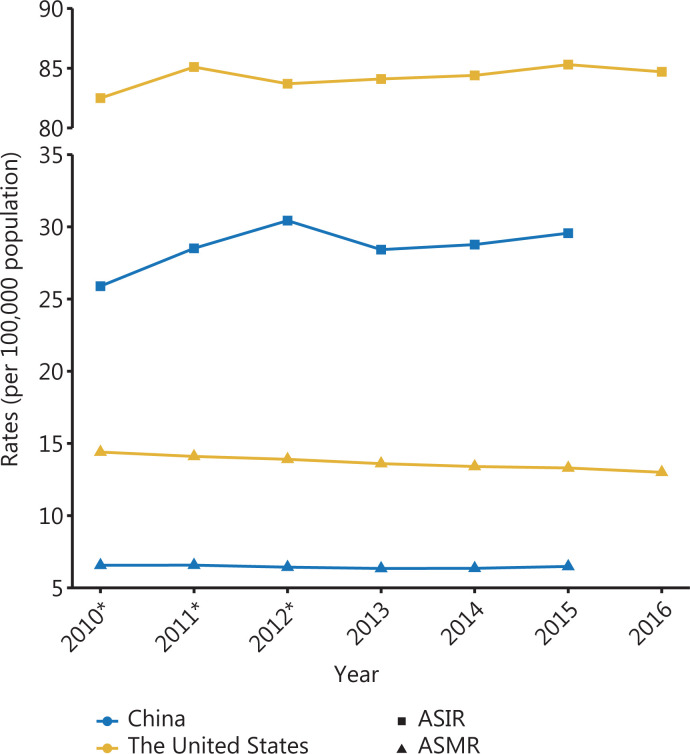
Epidemiological changes in breast cancer. The incidence and mortality of breast cancer in the United States and China. *The incidence and mortality rates of China in 2010–2012 are age-standardized rates according to China’s population in 2000. The others are age-standardized rates according to the world standard population (Segi’s population). ASIR: age-standardized incidence rates; ASMR: age-standardized mortality rates.

The United States and China have distinct trends in breast cancer incidence and mortality. Using nationwide statistics of cancer incidence and mortality from the International Agency for Research on Cancer and National Central Cancer Registry of China^2,19–24^, we plotted the changes in breast cancer incidence and mortality in recent years in the United States and China. As illustrated in **Figure 1**, the incidence of female breast cancer has plateaued and is slowly increasing in the United States. One explanation for this finding is that widespread breast cancer screening with mammography at the end of the last century has reached a plateau in the United States^5^. The decrease in the use of hormone replacement therapy after menopause has also contributed to the stabilization of the incidence^25^. Besides, mortality due to breast cancer in the United States began to decrease with the standardization and popularization of breast cancer screening, as well as advances in treatment (**Figure 1**). Compared with those in the United States, the breast cancer incidence and mortality rates in China continue to rise (**Figure 1**). Therefore, China must establish a standardized breast cancer screening and early diagnosis system for early detection of breast cancer to reduce mortality.

**Figure 2:**
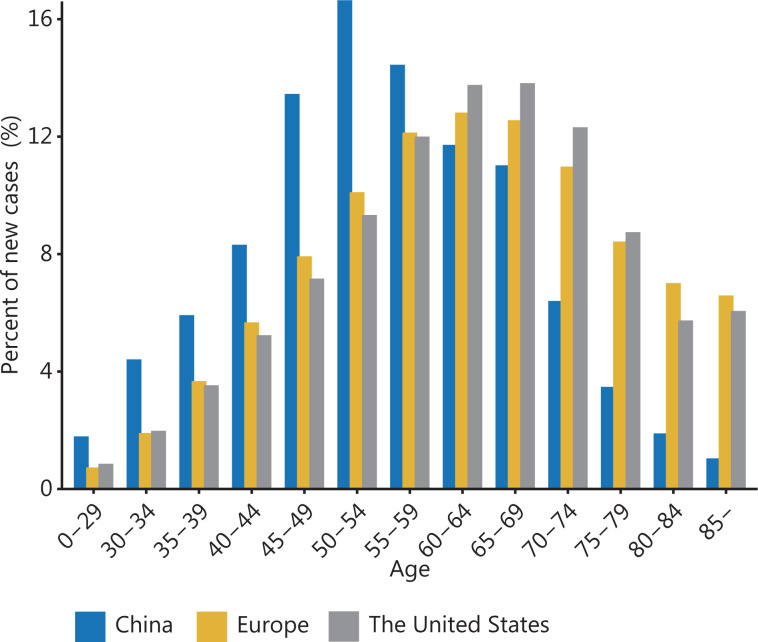
Age distribution of patients with breast cancer. Age distributions of patients with breast cancer in China compared with Europe and the United States. The data are from GLOBOCAN 2020.

Compared with patients with breast cancer in Europe and the United States, Chinese patients have distinct features in the age of onset. Our previous research has revealed 2 age peaks of breast cancer onset in China: one at 45–55 years of age and the other at 70–74 years of age^26^. The former peak is greater, thus resulting in a young median age of onset. According to the GLOBOCAN 2020 estimates, the median age of onset in China is 50–54 years, which is much younger than the 60–64 years in Europe and the United States (**Figure 2**)^27^. An earlier median age of onset suggests that more women have breast cancer at younger ages in China. Consequently, patients require longer follow-up and treatment periods, and have fewer healthy life years, thus increasing the disease burden for both individuals and society. Therefore, Chinese women must start breast cancer screening at an earlier age to enable early detection of suspicious lesions, improve prognosis, and reduce the burden of disease.

In summary, the incidence and mortality of breast cancer in Chinese women has been increasing, and the median age of onset is young, thus suggesting that breast cancer screening in Chinese women must be standardized and started earlier. The specific risk factors leading to these onset characteristics in Chinese women are also worthy of exploration.

### Risk factors for breast cancer in China

In China, a significant factor underlying the rapid increase in breast cancer incidence in recent decades is the decrease in the fertility rate among women^16^. Since the family planning policy was enacted in the 1970s, the national total fertility rate (TFR) decreased dramatically from approximately 6 in the 1960s to 1.22 in 2000. The TFR has since increased to approximately 1.3, owing to relaxation of the policy^28^. The fertility rates in Northeast China, Beijing, and Shanghai are lowest, with a TFR of only approximately 0.7. Correspondingly, the incidence of breast cancer in Northeast China is highest: the age-standardized incidence rate by the world standard population (ASIRW) is 35.4/100,000, whereas the total ASIRW is 28.7/100,000. In addition, significant differences in TFR exist between urban and rural areas (0.88 and 1.44, respectively)^29^. The ASIRW of breast cancer is also significantly higher in urban than in rural areas (33.8/100,000 *vs.* 23.6/100,000)^2^. Therefore, according to both the temporal and spatial distribution, the decline in fertility rate is accompanied by an increase in breast cancer incidence. Moreover, the incidence of breast cancer is higher in regions with significantly lower fertility rates, thus suggesting that the decline in fertility rate is an important reason for the increase in breast cancer incidence in China. In addition to policy restrictions, later ages at marriage and postponement of childbearing are important reasons underlying the decrease in the TFR. Late marriage and pregnancy in China are attributed to the lifestyle changes resulting from the rapid development of Chinese society since the 21st century. Women who have more education, have higher labor participation, and pursue career development may choose marriage and pregnancy at older ages^30^. Therefore, these women have lower fertility rates and higher incidence of breast cancer, particularly in large cities such as Beijing and Shanghai.

In contrast, some risk factors in China are similar to those in Western countries, including reproductive and hormonal factors, as well as dietary factors. Early menarche, late maternal age at the birth of the first or last child, and long intervals between menarche and the birth of the first or last child are all associated with higher breast cancer risk, particularly in premenopausal women, whereas a lower rate of breastfeeding and late menopause are important risk factors for postmenopausal women^31–33^. In addition, hormone replacement therapy and oral contraceptives contribute to the development of breast cancer, but their effects are not as substantial as those of reproductive factors^34^. Moreover, risk factors associated with diet, obesity, and exercise, which have been recognized as crucial contributors to the increased incidence of breast cancer in Western countries, warrant more attention in China^35^. Recent data have shown a substantial increase in the prevalence of obesity in China in recent years and indicated that a “meat-sweet pattern” diet increases breast cancer risk in postmenopausal Chinese women^36,37^.

In conclusion, breast cancer has distinct prevalence characteristics and risk factors in China. Consequently, targeted screening and early diagnosis strategies must be established for Chinese women.

## Imaging techniques for screening breast cancer

Screening for breast cancer mainly relies on imaging techniques, most importantly mammography, ultrasound (US), and magnetic resonance imaging (MRI). The features, advantages, limitations, and performance of these imaging techniques are summarized in **Table 1**.

**Table 1:** Comparison of common screening techniques

Technique	Current recommendations	Advantages	Disadvantages	Sensitivity (%)	Specificity (%)	Recall rate (%)	PPV (%)	Cancer detection rate (per 1000)	Ref.*
Mammography	Recommended for women who have reached the initial age for breast cancer screening	Convenient; economical; sensitive to microcalcification	Insensitive to high density breasts and deep lesions	69.0–86.0	57.0–96.6	3.5–4.0	13.0–22.9	3.2–7.1	^38–42^
DBT	Recommended, particularly for women with dense breasts, replacing DM	3D imaging, reducing tissue overlay	Greater radiation dose, examination time and cost	82.6–89.0	72.0–97.6	3.1–3.9	20.7–21.4	4.6–9.4	^38–42^
CEM	Recommended for women at high risk	Vascular functional imaging	Use of contrast agents; greater radiation dose	87.5–92.7	67.9–93.7	–	11.9–20.9	13.1–15.5	^43–45^
Ultrasound	Recommended, particularly for women with dense breasts, and pregnant and lactating women	Noninvasive; real-time; no radiation; elastography; identification of cystic and solid masses	Dependent on technologist experience; insensitive to lesions without clear mass	80.0–90.6	81.0–94.5	4.1–6.9	3.0–6.6	4.4	^46–48^
ABUS	Recommended, particularly for women with dense breasts, and pregnant and lactating women	Less dependent on technologist skill; reproducible	Unable to assess the axillary lymph node status	67.6–99.8	74.6–91.6	1.5–13.5	4.1–5.4	1.8–15.1	^46–48^
MRI	Recommended for women at high risk	Most precise identification of soft tissue; reflects both the anatomical structure and lesions; displays small lesions, multifocal lesions, and lesions located deep in the tissue	Insensitive to calcification; expensive; long examination time; use of contrast agents	95.2–97.5	83.8–92.0	9.5	8.0–17.4	11.8–16.5	^49–51^

DBT, digital breast tomosynthesis; DM, digital mammography; CEM, contrast-enhanced mammography; ABUS, automated breast ultrasonography; MRI, magnetic resonance imaging; PPV, positive predictive value. *Most relevant studies with evidence grade IIb or above are included to illustrate the performance of most imaging techniques, which were essentially the results of randomized controlled experiments or systematic review and meta-analysis.

### Mammography

#### Conventional mammography

Mammography, the most widely used screening technique for breast cancer, provides a two-dimensional projection image of the breast. In general, mammography is performed with craniocaudal and mediolateral views of each breast^52,53^. Mammography has developed rapidly in recent decades, and digital mammography (DM) has gradually replaced conventional screen-film mammography.

Because of its recognized ability to enable the diagnosis of breast cancer, as well as its high sensitivity to microcalcifications in early breast cancer, convenience, and economical clinical application, mammography is now the most important method for the screening and early diagnosis of breast cancer, and the preferred method in various guidelines^3,54^. Many meta-analyses have confirmed the efficacy of mammography. Since the application of mammography, the breast cancer mortality rate has decreased by approximately 20% in women over 40 years of age, and the effect is particularly significant in the older population, with a decrease of greater than 30% in women over 60 years of age^3,4,54,55^. However, limitations of mammography have also been observed. The sensitivity of mammography is relatively lower for women of young age with dense breasts^40,49^. In addition, overdiagnosis might increase with the increasing diagnostic rate of breast cancer in younger women with the widespread use of mammography^54,56^.

Many new techniques have been developed and popularized in clinical practice to further improve the sensitivity and specificity of mammography, and minimize overdiagnosis.

#### Digital breast tomosynthesis (DBT)

DBT, also called 3D mammography, acquires a series of stacked images while the X-ray tube is pivoted in an arc in a plane aligned with the chest wall^57^. With postprocessing algorithms, 2D mammogram images are reconstructed from tomosynthesis images in synthetic mammography, which is equivalent to DM.

This 3D imaging substantially decreases tissue stacking, thus enabling the discovery and localization of lesions hidden in high-density glands^58^. In DBT, compared with DM, the cancer detection rate is relatively higher in patients with high breast density or heterogeneous density^59,60^. Screening examinations with DBT are associated with a higher cancer detection rate and sensitivity, as well as a lower recall rate, thus decreasing false-positive rates and overdiagnosis^39,42,59,61^. In general, DBT may be a better choice than DM for breast cancer screening.

Although DBT performs better than DM for breast cancer screening, researchers have not conclusively determined whether DBT can replace DM, owing to its higher costs, radiation dosages, and acquisition and interpretation times^62^.

#### Contrast-enhanced mammography (CEM)

CEM is another emerging mammography technique that uses intravenous iodinated contrast agents to improve the visualization of tumor neovascularity. Dual-energy digital mammography provides a low-energy image, similarly to DM, and a high-energy image to detect the signal from the contrast agent^63^. The high-energy and low-energy images are postprocessed to generate the final recombined image of enhanced lesions in the breast.

With CEM, abnormal anatomical signs and the blood supply within lesions can be observed in the recombined images. Therefore, screening with CEM significantly increases the cancer detection rate in patients with dense breasts, and has a significantly higher sensitivity and specificity than screening with DM. No added benefit of using supplementary US examination has been shown^44,45^. CEM is only slightly less sensitive than MRI, but it provides higher specificity and a positive predictive value, as well as a similar ability to assess lesion size and disease extent^43,64–66^. Thus, in the future, CEM may be used as an alternative to MRI in breast cancer screening for women with moderate-to-high risk.

However, compared with conventional mammography, CEM has several disadvantages, including a greater radiation dose and risk of adverse reactions to contrast agents^67,68^. In addition, the limitations of 2D mammography images still apply, because some deep breast lesions cannot be visualized^63^.

### Ultrasound

Hand-held ultrasound (HHUS) is commonly used to examine breast diseases. One major advantage of US is its ability to distinguish breast cysts from malignant solid masses, thus helping avoid unnecessary biopsies^69^. Beyond conventional linear US, several advanced imaging techniques have been used to improve diagnostic ability. Color and power Doppler are important complementary tools for evaluating lesion vascularity. The demonstration of irregular branching vascularity and a greater number of peripheral vessels raises suspicion of malignant lesions^70,71^. US elastography can detect changes in the elasticity of soft tissues resulting from specific pathological processes. Higher lesion stiffness corresponds to a greater probability of malignancy^72^. In clinical practice, US is used mainly for supplementary screening in women with dense breasts and negative mammogram findings, and for localizing lesions identified by mammography or MRI for puncture biopsy^73,74^.

However, US also has limitations. First, the ability of US to indicate the nature of calcification is inferior to that of mammography^69^. In addition, US lacks standardized techniques and is time consuming. The diagnostic ability of US depends on the experience and ability of technologists, who must recognize lesions and thoroughly evaluate them through real-time scanning^75^.

Automated breast ultrasound (ABUS) is an advanced ultrasonography technique with high-frequency broadband transducers that provides 3D reconstructed images based on 3–5 views of each breast^76^. ABUS separates the acquisition step from interpretation, thereby prolonging the interpretation time. However, ABUS is less dependent on the technologist’s experience than HHUS, and computer-aided detection software for ABUS has been developed for ease of interpretation^77^. ABUS has high sensitivity and specificity comparable to or better than that of HHUS, and it outperforms HHUS in the detection of architectural distortions^78–82^. Therefore, ABUS may be a promising early screening method replacing HHUS, particularly in settings lacking skilled technologists.

### MRI

MRI is the most sensitive imaging technique for breast cancer screening. During the examination, women lie in the prone position with the breasts hanging free in the recesses of the coil, to allow the breast tissue to spread and minimize motion artifacts induced by breathing^83^.

The MRI approach currently used for breast cancer screening is multiparametric breast MRI, which provides better differentiation between benign and malignant lesions. T1-weighted contrast-enhanced sequences are the basis for MRI protocols to illustrate vascularity within lesions^84^. Time-signal intensity curves are highly valuable in assessing benign and malignant lesions, because malignant lesions exhibit a fast initial phase and rapid washout^85^. Ultrafast breast imaging is an advanced method that captures the fast early enhancement of malignancies within shorter acquisition times^86^. T2-weighted imaging performs well in revealing edema, a potential sign of malignancy^87^. Malignancies also show decreased water diffusion in diffusion-weighted imaging compared with normal tissue, thus leading to a higher signal intensity and a lower apparent diffusion coefficient^88^. In summary, multiparametric breast MRI provides high accuracy for the diagnosis of breast disease, and it has a better ability to detect invasive cancer than ductal carcinoma *in situ*^49,84^. Moreover, breast MRI can be used for multiplanar imaging of the breast, which specifically displays both the anatomical structure and the lesions. Small lesions and multiple scattered lesions are relatively easier to observe in MRI images, thus avoiding missed detection. Compared with DM alone or in combination with US, MRI substantially improves the sensitivity and cancer detection rate, and decreases interval cancers, particularly in women with dense breasts^49–51,89^.

However, the high sensitivity of MRI may come at the cost of low specificity and a high false-positive rate^50^. Therefore, MRI is currently clinically used for breast cancer screening in women at high risk to avoid missed tumors^90^. Long examination times and high costs are also important factors limiting the wide application of MRI in breast cancer screening. Abbreviated MRI, an emerging technique, uses a shorter image acquisition and interpretation time, thus increasing the availability of breast MRI and decreasing costs^91^.

As described, the various breast cancer screening techniques each have their own advantages and disadvantages, along with different detection abilities and indications. Therefore, standard evidence-based screening guidelines are needed to guide screening strategies for different populations.

## Establishment of a breast cancer screening strategy for Chinese women

### Breast cancer screening guidelines worldwide

The formulation of breast cancer screening guidelines requires data support from many studies to determine the screening techniques, the initial age of screening, and the frequency of screening. On the basis of the results of many large-scale screening studies conducted in Europe and the United States, several institutions have developed breast cancer screening guidelines.

For women with an average risk of breast cancer, among these guidelines, the American Cancer Society (ACS) breast cancer screening guidelines provide more active recommendations. The ACS strongly recommends that women start mammography screening annually at age 45 and transition to biennial screening at age 55 or continue screening annually. They also recommend that women 40–44 years of age be given the opportunity to begin screening^10^. However, other guidelines provide more conservative recommendations. The WHO and the European Breast Guidelines provide similar recommendations that women 50–69 years of age undergo biennial mammography screening^9,12^. The European Breast Guidelines also suggest biennial or triennial mammography screening for women 45–49 years of age and triennial mammography screening for women 70–74 years of age^12^. The U.S. Preventive Services Task Force (USPSTF) recommends biennial mammography screening for women 50–74 years of age. For women 40–49 years of age, mammography screening may yield false-positive results resulting in unnecessary biopsies and a higher risk of overdiagnosis. Therefore, the USPSTF recommends that women who place higher value on the potential benefits than the potential harms may choose to begin biennial screening at ages of 40–49 years^13^.

Regarding the selection of screening techniques, all these guidelines recommend mammography screening alone for women at average risk. The ACS and USPSTF recommend against breast self-examination for breast cancer screening, because evidence remains insufficient to assess the additional benefits and harms beyond mammography screening^10,92^. Moreover, both the USPSTF and the European Breast Guidelines conclude that insufficient evidence supports that additional screening with DBT, HHUS, or ABUS for women with dense breasts provides benefits outweighing the added costs. However, the European Breast Guidelines suggest DBT for women recalled for suspicious lesions on a mammogram^12,13^.

In contrast, the ACS recommends breast MRI screening for women with a high risk of breast cancer. Annual mammography and MRI screening are recommended starting at 30 years of age for women with *BRCA* germline mutation, women with a first-degree relative with a known *BRCA* germline mutation, and women with an over 20% lifetime risk of breast cancer, on the basis of risk estimation models^11^. Commonly used models for predicting an individual’s risk of breast cancer include BRCAPRO and BOADICEA, which focus primarily on family history and genetic factors, and the Tyrer-Cuzick and Gail models, which include classic risk factors^93^. Other conditions considered to be associated with a high risk of breast cancer include radiation to the chest at an age younger than 30 years; diagnosis of Li-Fraumeni, Cowden, or Bannayan-Riley-Ruvalcaba syndrome; and a personal history of breast lesions that are either malignant or benign^94,95^.

In summary, improved screening techniques, updated study evidence, and more detailed risk stratification will help establish better breast cancer screening guidelines.

### Imaging-based screening for breast cancer in Chinese women

In Chinese clinical practice, the selection of imaging-based screening was subjectively performed by clinicians referring to the screening strategies of Western countries, the breast features and personal history of patients, and facility conditions of hospitals^14^. Clinicians lacked standard screening guidelines to guide their screening selection. However, Chinese women have significantly smaller and denser breasts than white women, particularly women younger than 50 years^96,97^. Because previous studies have found that higher breast density affects the sensitivity and screening efficacy of some screening techniques, screening strategies suitable for Chinese women must be established.

US can be used as an adjunct to mammography in screening women with dense breasts^98,99^. Mammography is sensitive to microcalcification, but the sensitivity decreases significantly with increasing breast density. Lesions without calcification may be masked by dense fibroglandular tissue^100^. Compared with mammography, dense fibroglandular tissues are hyperechoic, and most lesions are hypoechoic on ultrasonograms. Therefore, US is not affected by higher breast density^101^. US may help identify the presence of an associated mass with suspicious calcification; its boundary, size, and shape; and the benign or malignant properties of lesions. US may also detect lesions on the edge or deep inside the breast, which may be missed on mammograms^102^. Rougher lesion margins and a lower lesion/glandular tissue stiffness ratio are often associated with lesions identified on ultrasonograms but not on mammograms^103^. Many international studies have shown that additional US with mammography for women with dense breasts improves sensitivity and increases the detection rate of cancer, particularly small, early-stage and lymph node-negative invasive cancer^89,104–108^. A randomized controlled trial in Japan has demonstrated that screening with mammography and ultrasonography has a significantly higher sensitivity than mammography alone (91.1% *vs.* 77.0%), with an incremental cancer detection rate of 1.7 per 1,000. Accordingly, the early diagnosis rate is increased, and interval cancer is decreased^107^. However, additional US screening may also decrease the specificity and increase the false-positive rates. This outcome is associated with indications for the selection of US^98^. A unified standard of indications for screening with US is not yet available.

Because Chinese women generally have smaller and denser breasts than women in Western countries, the FUSCC conducted a study to evaluate whether the combined use of US and mammography might be more suitable for Chinese women. A total of 14,464 women 35–74 years of age participated in this Shanghai Society-based Breast Screening Program. All participants first underwent a clinical examination, and those with positive results and all women 45–69 years of age underwent biennial mammography and US screening. US was found to be most sensitive in women younger than 45 years, whereas mammography performed better in older women. The combined use of US and mammography increased the sensitivity from 67.3% of mammography alone to 79.3%, particularly in hormone receptor-positive breast cancer. In addition, the study concluded that mammography plus US screening for women 45–69 years of age and mammography alone for women older than 70 years is a more cost-effective screening strategy. Given the large population and limited resources in China, clinical breast examination (CBE) is an effective screening method for younger women^109,110^. **Table 2** summarizes the results of several other studies on combined US and mammography screening for breast cancer in China^111–114^, all showing higher sensitivity for mammography plus US. Together, these findings indicate the effectiveness of supplementary US in Chinese women. However, this greater sensitivity comes at the cost of lower specificity and a lower positive predictive rate. The lower specificity may result from the separate interpretation of the mammogram and US images, which also leads to a higher recall rate. A combined assessment helps improve specificity^107^.

**Table 2:** Performance of US and MAM screening in programs in China

Study	Number of participants underwent screening	Number of breast cancer patients	Sensitivity (%)			Specificity (%)			PPV (%)		
			US	MAM	US + MAM	US	MAM	US + MAM	US	MAM	US + MAM
Ya-jie et al.^111^	11,236	54	57.4	79.6	92.6	–	–	–	–	–	–
Dong et al.^112^	31,918	99	61.6	84.8	94.9	98.8	98.1	97.2	13.6	12.2	9.5
Shen et al.^113^	4,135	14	100	57.1	100	99.9	100	99.9	70	72.7	60.9
Li et al.^114^	5,296	3,002	97.9	96.2	99.4	49.7	39.2	24.8	59.8	54.7	50.3

PPV, positive predictive value; US, ultrasound; MAM, mammography.

In summary, the combined use of US and mammography can be a suitable strategy for Chinese women, particularly younger women and women with dense breasts. For women 45–69 years of age, this choice is a better and more cost-effective method. The screening guideline established in China have taken the above factors into account and recommend that women 45–69 years of age with average risk should undergo biennial mammography, and supplementary US screening after mammography should be performed in women with dense breasts. CBE is an effective method for early screening in Chinese women. More detailed screening strategies are discussed in the following sections.

### Genetic screening for breast cancer in Chinese women

Genetic screening is another important method for screening breast cancer. A screen of germline mutations is useful to identify populations at high risk of developing breast cancer. *BRCA1/2* germline mutations are the most frequent germline mutations found in breast cancer and are present in approximately half of all patients with a family history of breast cancer^115^. *BRCA1* germline mutation carriers have a 37.9% cumulative risk of developing breast cancer by the age of 70 years, and the risk is 36.5% for *BRCA2* germline mutation carriers, which is approximately 10 times higher than that in the general population^116^. **Table 3** summarizes different germline mutation frequencies of most frequently mutated genes between breast cancers in Chinese and white patients. Among all patients with breast cancer, approximately 5% carry *BRCA1/2* germline mutations, and this percentage is similar in Chinese and white patients with breast cancers^121,126^. *BRCA2* germline mutation is more frequently detected than *BRCA1* in Chinese patients with breast cancer^120,122^. Approximately 3% of patients carry other germline mutations. *PALB2* is present at a relatively high frequency of 1%^118^. *ATM* and *CHEK2* are mutated more frequently in white breast cancer patients^117,119,120^. However, *RAD51D* germline mutation is more frequently present in Chinese patients and is particularly enriched in K91fs variants^127–129^. In addition, *RAD51D* germline mutation confers an increased breast cancer risk: carriers have a 20% cumulative risk of developing breast cancer by 80 years of age^129,130^. *PTEN* germline mutation is relatively rare in the Chinese population and is not recommended for genetic testing^125^.

**Table 3:** Comparison of frequent germline mutations between breast cancers in Chinese and white patients

Gene	Mutation frequency (%)		Ref.
	Chinese	White	
*BRCA1*	1.6–2.7	1.4–3.7	^117–123^
*BRCA2*	2.7–3.7	1.6–3.1	^117–123^
*PALB2*	0.7–1.0	0.9–1.0	^117–120,123^
*TP53*	0.3–0.5	0.2–0.3	^117,119,120,123,124^
*ATM*	0.4–0.6	0.7–1.0	^117,119–123^
*CHEK2*	0.2–0.3	1.6–2.1	^117,119–122^
*RAD51D*	0.4–0.5	0.1	^117,119,120,122^
*BARD1*	0.2	0.2	^117,119,120,122^
*PTEN*	0.1	0.1	^117,119,120,125^
*BRIP1*	0.2–0.3	0.2–0.8	^117,119,121–123^

FUSCC has developed an NGS-based pipeline for *BRCA1/2* mutation testing to better recognize possible *BRCA* germline mutations in patients with breast cancer and to alert affected relatives. The study has identified 5 risk criteria: TNBC, male breast cancer, bilateral breast cancers, early onset of breast cancer (diagnosed in patients younger than 40 years of age), and a family history of breast or ovarian cancer. Genetic testing is strongly recommended for patients with both a family history and either bilateral breast cancer or male breast cancer, as well as for patients with family history of at least 3 affected relatives. Moderate recommendations have been provided for patients with any 2 of 5 risk criteria or patients with family history of at least 2 affected relatives, and low recommendations have been provided for patients with any one of 5 risk criteria^126^. This pipeline has been applied in clinical diagnosis to recognize the genetic risk factors for patients with breast cancer and their relatives. Moreover, because its high cost makes DNA sequencing inaccessible for most people, FUSCC launched and developed an NGS panel called Fudan-BC panel, including 484 breast cancer-specific genes that are frequently mutated and are potential therapeutic targets in breast cancer. This panel aids in the preliminary detection of high-frequency germline mutations in more patients^131^.

Overall, the identification of the genomic characteristics and mutation profiles of breast cancer in China enables the identification of high-risk populations of breast cancer and the determination of indications for genetic testing. Moreover, genetic testing plays an important role in breast cancer risk stratification and screening.

### Guidelines for stratified screening for breast cancer in Chinese women

In addition to breast density and genetic factors, many other risk factors for breast cancer have been identified, as described above, including a history of benign breast disease. Stratification of these risk factors is important for the establishment of breast cancer screening strategies. The Gail model is widely used in Western countries. However, the predictive performance of this model in China is limited, because it may overestimate the risk in Asian women^132^. Therefore, a risk prediction model suitable for Chinese women is needed. FUSCC has developed a risk prediction model for breast cancer by analyzing information on various risk factors collected through a questionnaire, including family history and personal characteristics. This model has high predictive value, with an accuracy of 64.9%, a sensitivity of 79.0%, a specificity of 64.8%, and an area under receiver operating characteristic curve of 0.762^133^. Other researchers have built risk prediction models with an expected/observed ratio of nearly 1.0 and an area under the receiver operating characteristic curve exceeding 0.6^134,135^. These risk models could be applied for self-assessment and large-scale preliminary screening for breast cancer, but they would still require further improvement and exploration before being widely applied in clinical practice in the future.

**Figure 3:**
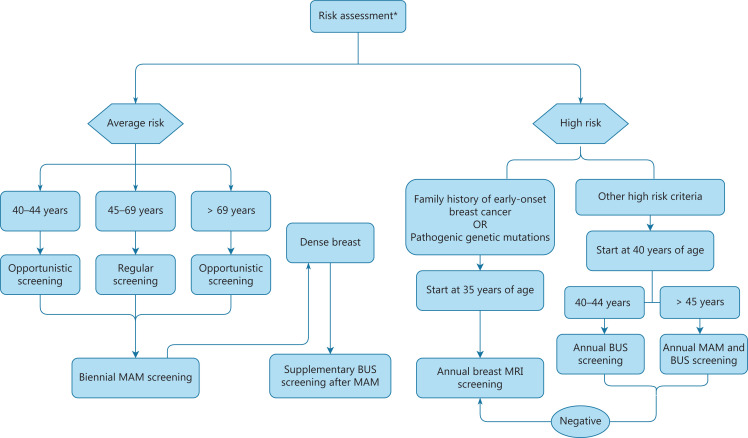
Risk stratification guidelines for breast cancer screening. Risk stratification flowchart for selecting breast cancer screening methods. *Women meeting any of the following criteria are considered to have a high risk of breast cancer: (1) family history of early-onset breast cancer, male breast cancer, pathogenic genetic mutations, or hereditary tumor syndrome; (2) at least 2 first-degree relatives with breast cancer, ovarian epithelial cancer, fallopian tube cancer, or primary peritoneal cancer; (3) personal history of moderate to severe dysplasia or benign breast cancer; or (4) history of chest radiotherapy^136^. MAM, mammography; BUS, breast ultrasonography.

According to the characteristics of breast cancer in Chinese women described above and with reference to international screening guidelines, the China Anti-Cancer Association has formulated breast cancer screening guidelines for Chinese women^136^. The recommended screening process is shown in **Figure 3**. Compared with other international guidelines, this guideline has several important features. First, because the peak age of breast cancer in Chinese women is younger than that in Western countries, the guideline recommends an earlier initial age of screening. Women 45–69 years of age with average risk should undergo biennial mammography, and women 40–44 years of age should undergo opportunistic screening. Second, because of the large population and limited resource settings in China, CBE is a preliminary screening method that can be used before imaging screening, particularly among women older than 50 years. The screening interval is biennial for women with an average risk of breast cancer. Moreover, the guidelines have additional recommendations for the use of US in breast cancer screening. Because of the high breast density in Chinese women, supplementary US screening after mammography in women with dense breasts is recommended. US is also recommended for women with a high risk of breast cancer but without genetic factors, such as US alone for women 40–44 years of age and US combined with mammography for women older than 45 years. This guideline also provides specific recommendations for women with high breast cancer risk. The guidelines divide high-risk women into 2 groups: those with a high-risk family history of breast cancer or pathogenic germline mutations, and those with other high-risk factors, such as a history of benign breast diseases or radiation therapy applied to the chest. MRI is primarily recommended for screening in the former group, whereas US and mammography screening are mainly recommended in the latter group, because this practice is more cost-effective^136–138^.

The establishment of breast cancer screening guidelines suitable for Chinese women has standardized the screening process in China, thereby improving the detection rate and early diagnosis of breast cancer, and the long-term survival rate of Chinese patients with breast cancer.

## Achievements and prospects

**Figure 4:**
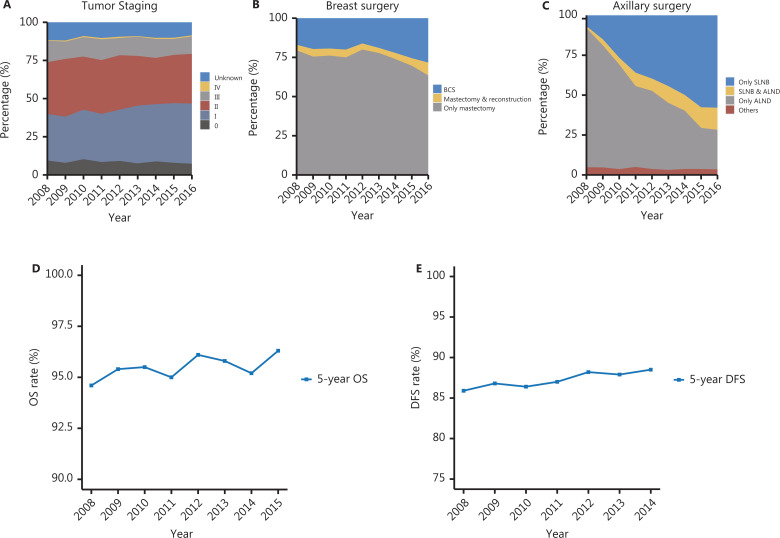
Improvements in early diagnosis and prognosis at FUSCC. (A–C) Trends in tumor staging at diagnosis (A), breast surgical modality (B), and axillary surgical modality (C) of breast cancer from 2008 to 2016 at FUSCC. (D, E) Trends in the overall survival rate from 2008 to 2015 (D) and disease-free survival rate from 2008 to 2014 (E) in patients with breast cancer treated at FUSCC. BCS, breast-conserving surgery; SLNB, sentinel lymph node biopsy; ALND, axillary lymph node dissection; OS, overall survival; DFS, disease-free survival.

Breast cancer screening has important effects on the diagnosis and treatment of breast cancer. Through the standardized application of breast cancer screening methods recommended by the guidelines, the FUSCC has made substantial achievements in breast cancer screening and early diagnosis. The stage of cancer at diagnosis decreased from 2008 to 2016. According to the TNM staging system, the percentage of patients diagnosed with stage I disease increased significantly, from 30.5% to 39.4% (**Figure 4A**). In addition, the number of patients undergoing breast-conserving surgery increased from 17.0% in 2008 to 28.3% in 2016, and the percentage of patients opting for reconstruction after mastectomy increased from 3.5% to 8.2% (**Figure 4B**). Moreover, with the development of sentinel lymph node biopsy (SLNB), more patients can preserve axillary lymph nodes after biopsy. The percentage of patients undergoing only SLNB markedly increased from 6.9% in 2008 to 57.9% in 2016 (**Figure 4C**). Importantly, the early detection of breast cancer and the use of less invasive surgical modalities have increased the long-term survival rates among patients. The 5-year overall survival rate of patients treated at FUSCC increased from 94.6% to 96.3%, and the 5-year disease-free survival rate increased from 85.9% to 88.5% (**Figure 4D, 4E**). In conclusion, standardized breast cancer screening reduces the burden of breast cancer treatment and substantially improves the survival and prognosis of patients with breast cancer.

Over the past 20 years, breast cancer screening in China has begun to receive attention and has gradually been standardized, thus enabling the achievements described above. In this development process, progress in science and technology has played a crucial role in promoting breast cancer screening. Updated screening techniques make breast cancer screening more accurate and convenient. Advanced mammography techniques such as DBT and CEM have substantially improved the sensitivity for detecting malignancies in dense breasts. ABUS has enabled US screening to become more convenient and universal. New MRI techniques have also simplified the MRI screening procedures. The advances in, and comprehensive use of, these screening techniques have greatly improved the sensitivity and specificity of cancer detection, and increased the accuracy of distinguishing benign from malignant lesions. Future technical progress is also expected to minimize the existing disadvantages, such as the harm due to use of radiation and contrast agents, to better benefit the public. The development of high-throughput sequencing technologies will allow for simultaneous sequencing of multiple pathogenic genes and even the whole exome or the entire genome of a patient^139^. Although the current cost of such sequencing remains high for the public, genetic testing will probably be popularized, and precise screening is a likely future trend for screening. The application of artificial intelligence (AI) is another trend expected in future screening. AI systems can assist radiologists in image interpretation, thus substantially improving the accuracy. Many studies have shown that AI systems simultaneously improve sensitivity and specificity, decrease false-positive and false-negative rates, more accurately identify invasive cancer, and are robust for breast cancer screening^140–145^. Furthermore, AI systems may be used to predict the personalized risk of breast cancer. The assessment of personalized risk prediction is based on combined assessment of an individual’s risk factors and breast images^146–149^. This approach facilitates risk stratification for breast cancer screening and thus guides the use of appropriate screening methods for different women. In addition, with greater understanding of tumor molecular characteristics, some tumor genomic and epigenomic features may serve as biomarkers for breast cancer. In this regard, liquid biopsy to detect these features may become an important method for breast cancer screening^150,151^. Combined with advances in multiomics studies, breast cancer screening and recognition are expected to become more precise.

China is continually updating and refining breast cancer screening guidelines suitable for Chinese women. In addition to technological innovation, more large-scale cohort studies in the Chinese population are needed to obtain additional evidence and establish better screening strategies suitable for the Chinese population. Furthermore, standardized breast cancer screening will substantially increase the survival of patients with breast cancer in China and reduce mortality.

## Conclusions

Breast cancer screening and early diagnosis in China are being standardized and popularized. This review highlights the background and the establishment of breast cancer screening strategies in China. The unique demographic and genetic characteristics of breast cancer in China summarized in this review have provided an important basis for the Chinese breast cancer screening guidelines. This knowledge will help guide future related studies and further improvement of breast cancer screening guidelines in China. Evidence-based and standardized breast cancer screening programs will play important roles in improving the prognosis of patients with breast cancer.
